# Eliciting preferences for attributes of Newcastle disease vaccination programmes for village poultry in Ethiopia

**DOI:** 10.1016/j.prevetmed.2018.08.004

**Published:** 2018-10-01

**Authors:** Z.G. Terfa, S. Garikipati, G. Kassie, J.M. Bettridge, R.M. Christley

**Affiliations:** a Management School, University of Liverpool, Chatham Street, Liverpool L69 7ZH, UK; b ICARDA, ICARDA c/o ILRI, P.O. Box 5689, Addis Ababa, Ethiopia; c Institute of Infection and Global Health, Department of Epidemiology and Population Health, University of Liverpool, Leahurst Campus, Chester High Road, Neston, Cheshire CH64 7TE, UK

**Keywords:** Poultry, Newcastle disease, Vaccine, Preference, Choice experiment

## Abstract

Newcastle disease (NCD) is an important disease of poultry, directly affecting the livelihoods of poor farmers across developing countries. Research has identified promising innovations in NCD vaccine development and field trials among village poultry have been promising. However, NCD vaccination is not currently part of village poultry extension programmes in many developing countries. Understanding the preferences for, and relative importance of, different attributes of potential vaccination programmes to prevent NCD will be crucial in designing acceptable and sustainable prevention programmes. This research employed the discrete choice experiment approach to elicit farmers’ preference for attributes of NCD vaccination programmes for village poultry in rural Ethiopia. The choice experiment survey was conducted on 450 smallholder farmers. The relative importance of attributes of NCD vaccines to farmers was estimated using a random parameter logit regression model. The preferred NCD vaccine programme had greater bird-level protection (i.e. greater capacity to reduce mortality should NCD occur in a flock), was delivered by animal health development agents, and could be administered via drinking water. Results from simulations on changes in attribute levels revealed that bird-level protection capacity and delivery of vaccine by animal heath extension affect farmers’ preferences more than other attributes. These findings suggest that it is important to ensure NCD vaccine programmes offer reasonable capacity to protect against mortality. It also suggests the need to understand farmers’ preferred vaccine delivery mechanisms and route of vaccine administration for a wider acceptance of vaccine.

## 1 Introduction

Livelihoods in rural Ethiopia typically depend on a complex array of small on-farm and off-farm enterprises. By virtue of the requirement for low start-up capital and relatively undemanding management skill, poultry^1^ production is the most widespread livestock enterprise in rural Ethiopia. Village poultry production has the potential to provide food and income and is an important component of food security for the rural poor. However, infectious and parasitic diseases affecting poultry among many other things, remain important constraints to poultry production, and to the realization of poultry’s potential for food security in many developing countries.

Disease and poor management have been emphasised as the major limitations to chicken production (Henning et al., 2009). In particular, Newcastle disease (NCD) is considered as one of the most important poultry disease (FAO, 2014). In rural Ethiopia, NCD is widespread (Dessie and Ogle, 2001; Zeleke et al., 2005) and believed to cause high mortalities. Where successful, control of NCD through routine vaccination has greatly reduced the impact of this disease in village poultry (FAO, 2014). However, implementation of NCD vaccine programmes to village chickens in developing countries, particularly in sub-Sahara African countries, is limited. One of few examples of implementation of an effective NCD control programme in African countries is that of Mozambique, which, in combination with improved management strategies resulted in increased chicken stocks, improved households’ food security and access to nutritious food and empowered women (Bagnol, 2001; Woolcock et al., 2004).

The potential benefits of village poultry to food security will remain unrealised whilst poultry die in vast numbers annually, from preventable diseases such as NCD. This is especially the case when village poultry producers remain disengaged from national animal health services (Alders, 2014). Hence, while introduction of improved genetic material will play an important role in transforming the traditional village poultry production in Ethiopia (as well as in other developing countries), such gains may be greatly limited by disease like NCD. Therefore, access to efficient and acceptable poultry health services, incorporating effective preventive measures, become essential.

The challenges of Newcastle disease control in endemic areas are very different in village, compared to in the intensive, production systems. In the intensive poultry industry, prevention is achieved by repeated applications of suitable vaccines. Such control methods which are relatively expensive and require appropriate vaccine storage conditions are neither feasible nor affordable in rural areas (Ideris et al., 1990). Under village poultry settings, an effective NCD control programme must be applicable in the absence of a viable cold chain (Alders, 2014), and vaccine must be deliverable in small quantities (Bensink and Spradbrow, 1999).

Some existing poultry vaccines can be used under these less than optimal field conditions, and they offer the means for farmers to protect their flocks (McLeod and Rushton, 2007). Thermostable NCD vaccines, developed over the past few decades have been used effectively in village settings. Implementation of the heat tolerant V4 vaccine (Spradbrow et al., 1988) has provided promising results in some African countries (Alders and Spradbrow, 2001). The I-2 NCD vaccines, produced in freeze-dried form, maintain its activity for eight weeks when stored below 30 °C (Alders, 2003). Studies on the application of these vaccines, usually under trial conditions, in village poultry across developing countries have demonstrated that it is possible to effectively control NCD in these settings (Wambura et al., 2000; Copland and Alders, 2005; Msoffe et al., 2010). More recently Lal et al. (2014) reported the development of low-dose, fast-dissolving tablet vaccines, each containing up to 50 doses of vaccine, that could maintain virus stability for more than six months at 4 °C. This allows for compact and better packaging and hence it could provide a promising option to control NCD in village poultry across developing countries.

Despite the development of thermostable NCD vaccines for village poultry and the need to control NCD in village chickens, it has proved difficult to achieve a sustainable control programme. Implementation of a successful and sustainable NCD control programme should incorporate economic sustainability, based on the commercialisation of the vaccine and vaccination services, and delivery of effective extension materials including methodologies among others (Alders et al., 2010).

We contend that it is imperative for Ethiopia, a country where egg and chicken are predominantly supplied from village poultry, to have a well-designed policy for NCD control in rural areas. There exists, within Ethiopia, the capacity to produce millions of doses of NCD vaccines (Anebo et al., 2014) and NCD vaccines have routinely been provided to commercial poultry producers. However, currently there is no comprehensive policy to control NCD in village poultry. The sustainability of effective NCD vaccination programmes will depend on prevailing production systems, poultry keepers’ preference for vaccine technology and alternative approach to deliver the vaccine. However, there is currently no information available regarding poultry keeper’s preference for vaccine programmes. This study, therefore, aimed to evaluate farmers’ preference for attributes of possible NCD vaccine programmes.

## 2 Materials and methods

### 2.1 Study area

This study was conducted in the Horro district of Central Western Ethiopia. Poultry is consumed during festive periods and has significant social importance in the community. Nonetheless, farmers in this area keep poultry primarily for sell of chicken and egg (Terfa, 2015). The district was, therefore, selected because of the high potential for poultry to contribute to the livelihoods of rural households in this region. Furthermore, previous work with farmers in the area has identified infectious disease, including NCD, as major impediments to poultry production (Dessie and Ogle, 2001). The mainstay of life in the district is rain-fed mixed crop-livestock farming system. The district receives average annual rainfall of 1685 mm (ranging from 1300 to 1800 mm) and annual temperature of 19 °C (ranging from 14 to 24 °C) (Desta et al., 2013). Major livestock species kept by farmers in this area include cattle, sheep, poultry, and goat, while the main crops grown include wheat, *teff*, maize, barley, and beans.

### 2.2 Discrete choice experiment designing and survey

Preferences for village poultry NCD vaccination programme were elicited by using a Discrete Choice Experiment (DCE) approach. The discrete choice experiment survey reported here involved several design phases. The process began with the collection of expert opinion on the attributes and attribute levels of potential NCD vaccination programmes. Initial findings were explored through discussion with experts in poultry health and examination of research literature to validate the identified attributes and attribute levels. Subsequently, two focus-group discussions were conducted in the study area, involving farmers and livestock development and marketing agency workers, to gauge the practicality of communicating the identified attributes and attribute levels to farmers. This process identified five attributes and their levels for the choice experiment. From the focus group discussions, it was learnt that farmers understood the risk of bird’s death due to NCD to be a two-part process; a risk that disease occurs in the flock and the risk, when it does, of one or more birds dying. This lead to the decision to include two attributes related to NCD protection; ‘flock-level protection’ (that is, the ability of vaccination to prevent outbreaks within the flock) and ‘bird-level protection’ (that is, the ability of the vaccine to prevent bird deaths should a flock be affected by NCD). The other attributes were ‘route of vaccine administration’, ‘delivery mechanism’, and price. As the vaccine was available in 50 dose vials, which was more than sufficient to vaccinate even large village flocks, cost was calculated based on 1 vial per flock per administration and was expressed per flock per year, regardless of the number of birds in the flock. In addition, findings of previous study on willingness to pay for vaccine service, conducted in the same setting, (Terfa et al., 2015) was also used as reference to determine possible cost of vaccine. A summary of attributes and attribute levels used in the final designing process is presented in Table 1.

**Table 1:** Attributes and attribute levels in the choice experiment.

No.	Vaccine Attribute	Attribute level	Reference level
1	Flock-level protection	1 30 % 2 50 % 3 70 %	50 percent
2	Delivery mechanism	By vet. technician 1 By trained farmers	By trained farmer
3	Route of administration	1 in water 2 in feed 3 Aerosol Spray	Aerosol spray
4	Bird-level protection	1 20 % 2 40 % 3 60 %	40 percent
5	Price of vaccine	1 ETB 60.00 2 ETB 80.00 3 ETB 100.00	Used as continuous

For analysis of data, cost of NCD vaccination, three times in a year, was included in the model as a continuous variable with their actual levels. All other attributes of the designed NCD vaccine programme were treated as discrete variables. For each discrete attribute in the DCE with levels L, we created L-1 discrete variables. Following suggestion by Hensher et al. (2005), effects coding was used to measure nonlinear effects in the trait levels and to avoid confounding effects of reference levels in the grand mean. In effects coding, the reference level is coded as -1 unlike the dummy coding approach where the reference level is coded 0.

The unlabelled DCE was designed to produce NCD vaccine programme profiles using the identified attributes of vaccine. There are 162 (3^4^*2) possible ways to combine the attributes their levels which could be cognitively too challenging for respondents. Therefore, 36 profiles were created out of the 162 possible combinations by applying the SAS algorithm D-efficiency criterion. The final design had a D-efficiency of 99.8, suggesting that the variance matrix should generate reliable estimates. These profiles were randomly classified into 18 vaccine profiles, each choice set having two profiles, and blocked into three. Hence each respondent was presented with six choice sets, each containing two vaccine profile and opt-out option. An opt-out option was included to each choice set to avoid forced choice, so that the DCE is consistent with utility maximization and demand theory (Bateman et al., 2002). Choice sets were supplemented by visual aids (pictures) to help communicate information about attribute levels (sample choice cards will be provided as supplementary materials).

The study was approved by the University of Liverpool’s Committee on Research Ethics (VREC76). Prior to the formal survey, the questionnaire was piloted and pre-tested on 30 individual farmers and in focus group discussions consisting of 11 farmers (two female and nine male discussants) during early January 2013. The pilot survey for the DCE indicated that communicating attribute and attribute levels was workable and respondents could complete the choice exercise. Following the feedback from pilot survey, the questionnaire was reordered and presented in such a way to optimize respondents’ attention for the choice task. The formal survey was conducted in February and March 2013. The survey was conducted in four ‘*Gandas’*(the lowest administrative unit in government structure consisting of several villages). The four ‘*Gandas’* were selected by the project from two different market channels in the district. This study was part of bigger project that comprises study on genetics and epidemiology of village chicken diseases. Selection of the *’Ganada’* was done purposely to capture the diverse gene-pool and diseases prevalence from two different market channels. This DCE survey was administered to 450 farmers drawn from a list of farm households in the four ‘*Gandas’,* provided by local development agents, employing sampling with probability proportional to population size. The survey was conducted by well-trained and experienced enumerators with supervision and oversight by the researchers.

### 2.3 Modelling framework

We used the random parameter logit (RPL) model in the analysis presented here. It overcomes major limitations of conditional logit model (Train, 2009). In RPL, the utility that individual n obtains from alternative j in time (choice situation) t is given by

(1) $$\begin{array}{cc} U_{nit}=\beta x_{njt}+\left[\eta_{n}x_{nit}+\varepsilon_{nit}\right] & n=1,\ldots ,N,\;j=1,\ldots ,J,\;t=1,\ldots ,T \end{array}$$

Here, $\beta_{n}$ is the vector of mean attribute utility weights in the population whereas $\eta_{n}$ is the vector of person n-specific deviations from the mean; $x_{njt}$ is a vector of observed explanatory variables; and $\varepsilon_{njt}$ is assumed IID extreme value. The investigator may specify any distribution for the $\eta_{n}$ vector. In this research, a normal distribution was specified for attribute levels of ‘bird-level protection’ and ‘delivery mechanism’ and triangular distribution for attribute levels of ‘flock-level protection’ and ‘route of vaccine administration’, while the price was specified as fixed.

The choice probabilities in RPL are simulated using;

(2) $$P\left(j,x_{nt}\;\right)=\frac{1}{D}\sum_{d=1}^{D}\frac{exp[(\beta +\eta^{d})x_{nit}]}{\sum_{k=1}^{J}exp[(\beta +\eta^{d})x_{nkt}]}$$

Thus, given *D* draws, one could obtain simulated choice probabilities by averaging simple logit expressions over these draws. Hensher et al. (2005) suggest trying a range of draws starting from as low as 25 and going up until well-behaved models are fitted, to determine the number of draws for the simulation. In the present study, 200 Halton draws was used. The model was estimated using, econometric software, NLOGIT version 5.

## 3 Results

### 3.1 Farmers’ characteristics

Socioeconomic characteristics sample farmers are summarised in Table 2. The average age of the respondent farmers was about 42 years (median 38) and the average family size was 6 ± 2 persons. On average, farmers had one child below five years and the average number of children below 17 years was 4 ± 2. About 38% of farmers had attended elementary school and 16% of them had attended high school and 12% of them could read and write. However, a significant proportion of farmers (31%) had no formal education. About 80% of respondents were male farmers and 20% were female. This large gender disparity was observed because we targeted head of the household, majority of whom were male, based on the lists of farmers available for sampling.

**Table 2:** Respondents’ descriptive statistics and code used in the analysis.

Variables	Code /unit	Descriptive
Age	Years	Mean = 41.62 (SD = 14.87)
Family size	Number of persons within the family	Mean ± SD = 6 ± 2
Children below 5 years	Number of children	Mean = 1.1 (SD = 0.9)
Children below 17	Number of children	Mean = 3.6 (SD = 2.0)
Education	1= illiterate	31.3%
	2= read and write	12.0%
	3 = elementary	37.8%
	4 = secondary	16.0%
	5 = above secondary	2.9%
Sex	Male	80.4%
	Female	19.6%

### 3.2 Empirical result

The results from the simulated maximum likelihood estimates of random parameter logit (RPL) model, based on the analysis of DCE data obtained from 450 farmers survey, are reported in Table 3. The overall explanatory power of the model was high with a pseudo- $R^{2}$ of 0.43. The intercept in the model result representing the opt-out option in the alternatives provided for choice had a negative, and statistically significant, mean coefficient. This indicates a strong reluctance to opt-out such that respondents preferred to choose from the two alternatives associated with various attribute levels.

**Table 3:** Random parameter logit model result for NCD vaccine programme attributes using simulated likelihood estimation.

Variables	Coefficient	Standard Errors
Random parameters in utility functions		
Flock-level protection		
30 percent efficacy level	−0.132*	0.067
70 percent efficacy level	0.178**	0.074
Route of administration		
In water	0.374***	0.082
In feed	−0.133***	0.078
Vaccine delivery by		
Veterinary technicians	0.581***	0.093
Bird-level protection		
20 percent of chicken would survive	−1.652***	0.128
60 percent of chicken would survive	1.709***	0.140

Non-random parameters in utility functions		
Price of vaccine	−0.0007	0.003
Constant	−4.084***	0.337

Standard deviation of random parameters		
30 percent efficacy level	0.441	0.451
70 percent efficacy level	0.012	0.291
With water	1.493***	0.418
With feed	0.023	0.346
Veterinary technicians	0.474**	0.204
20 percent of chicken would survive	0.097	0.125
60 percent of chicken would survive	0.097	0.126
Number of respondents	450	
Number of observations	2,700	
Number of Halton draws(R)	200	
Log likelihood function	−1681.689	
Restricted log likelihood	−2966.253	
$x^{2}(df=21)$	2569.127	
McFadden Pseudo R-squared	0.433	

*Note*: ***, **, and * significant at α equal to 0.01, 0.05, and 0.1 using Wald statistics.

Results from RPL revealed preferred attributes of NCD vaccination programme attributes. The route of vaccine administration was also strongly statistically significant and had a positive mean coefficient for NCD vaccine if given in water, but a negative mean coefficient if given in feed. This may suggest farmers would prefer NCD vaccine that could be administered via water with a likelihood for more uptake, by farmers if the vaccine could be given in water. Route of vaccine administration affects vaccine efficacy; trial studies in village poultry show that I-2 vaccination administered via water is twice as efficacious as compared to vaccine administered via food carrier (Habibi et al., 2015). Therefore, future NCD vaccination programme could possibly achieve better efficacy and wider acceptance by using preferred route of vaccine administration, via water.

NCD vaccine programme that uses a veterinary technician (animal health development agent) for delivery of a vaccine was also statistically significant with a positive mean coefficient. This implies that farmers prefer NCD vaccine programme using a veterinary technician for delivery over one that would be administered by trained farmers. Previous reports on animal health service provider choice suggests a preference for village animal health workers and cooperatives, mainly because of their accessibility (Mirajkar et al., 2011; Lamichhane and Shrestha, 2012). In the study area used here, veterinary technician (animal health development agent) live within rural communities and hence accessibility was probably not a concern for farmers. Moreover, our research is to understand the preference for providers of a specific technical service - NCD vaccination - and hence providing the service by trained farmers was perhaps not considered as a viable alternative. In this study, trained farmers as a provider of NCD vaccination was included to explore possibility of developing community based approach in the future and to understand farmers’ preferences.

Result from RPL indicates that flock-level protection of the NCD vaccine programme was statistically significant, where higher flock-level protection (70%) had positive mean coefficient, but lower flock-level protection had negative mean coefficient. As expected, farmers exhibited a preference for NCD vaccine programme that has higher flock-level protection capacity. The RPL model result also revealed that bird-level protection was also statistically significant. Vaccine programmes with greater bird-level disease protection had a positive mean coefficient, suggesting farmers’ preference for an NCD vaccine programme that prevented mortality in the face of NCD outbreaks. The model result also revealed that price is not an important attribute of a vaccine programme profiles, as indicated by a statistically insignificant price coefficient. This implies that farmers tend to prefer better vaccine service, in terms of bird-level protection and delivery mechanism, but somewhat less concerned about paying for the service. A study in India by Ahuja et al. (2003), similarly, found that being charged for services is not an important determinant of the decision to use veterinary services and sharp declines in the use of these services as a result of reduction and removal of subsidy is unlikely.

The random coefficients for the attributes of vaccine delivery by veterinary technician (animal health development agent) and vaccine administration with water have highly significant standard deviations (Table 3). This implies that not all farmers attach equal weight to these vaccine attributes. The estimated means and standard deviations of the normally distributed coefficients could provide information about the share of the population that places positive values or negative values on the respective attributes or attribute levels (Train, 2009). Considering attributes with statistically significant standard deviation estimates in the model result, 87% of farmers had a positive preference for vaccine service that would be administered by veterinary technician while 13% of respondent had negative preference for this vaccine attribute.

Estimation of the RPL with various distributional assumptions and treatment of price as categorical variable were tried, to get a result where price would be significant. However, price was found to be statistically insignificant under all appropriate distributional assumptions and finally it was treated as a continuous and non-random variable. Nonetheless, price coefficient had a negative sign, as expected. A drawback of the statistically insignificant price variable RPL is the fact that estimation of economic worth of attributes of vaccine programmes is impossible. Profile simulation programme was also employed to investigate the marginal effect of change in attribute levels on the choice of alternatives. Studies in the past that found insignificant price coefficient also employed similar approach (see Kassie et al., 2009).

### 3.3 Simulations of changes in NCD vaccine attribute levels

We also employed profile simulation programme to investigate the marginal effect of change in attribute levels on the choice of alternatives. The simulation programme can be used to predict the set of choices for the sample and then examine how those choices would change if the attribute levels of the choices changed. Therefore, policy implications of changes in attribute levels in the present study were drawn from simulations with different attribute level scenarios assigned to each alternative. Various scenarios of vaccine profiles were identified by fixing attributes at different levels in each profile. The simulation results indicated the marginal effect of an attribute level on choice, which identifies the attributes most preferred and hence important to farmers.

The result for simulated changes in proportion of NCD vaccine profiles chosen is presented in Table 4. In our survey, each choice set had three alternatives; two NCD vaccine programme profiles and an opt-out option. In the survey, the base scenario, profile 1 was chosen in about 60% of the cases, profile 2 was chosen in about 39% of the cases and farmers opted-out in only 1% of cases. Of the various scenarios considered in the simulation, a significant change in the proportion of vaccine profile chosen was observed in a scenario where the attribute level for the vaccine’s bird-level disease protection capacity after an outbreak was fixed to 60% across all the choice sets for both profile 1 and profile 2. When it was set to 60% for profile 1, the simulation result indicated that the proportion with which profile 1 was chosen of all the choice sets would have increased by 17.3%. Similarly, the change in proportion profile 2 was chosen from all the choice set would have increased by 20.9% when bird-level protection capacity was set to 60% for profile 2. Another important simulation result was observed in a scenario where the vaccine service was set to be delivered by a veterinary technician. The proportion of cases in which profile 1 was chosen from all the choice set would have increased by 8.1% when the vaccine service was set to be delivered by veterinary technician for profile 1. When the same scenario was set for profile 2, the proportion chosen for profile 2 would have increased by 7%. The simulation results, generally, revealed that vaccine’s bird-level protection (i.e. capacity to reduce mortality from NCD) and the delivery mechanism are the two most important attributes of vaccine. These two attributes of NCD are, therefore, important aspects of NCD vaccine programme that would influence adoption of the technology. The reduction in the proportion of opting out observed under all scenarios indicates that farmers consider attribute levels in the choice exercise and choose vaccine profile with bundle of best attribute level, rather than opting out.

**Table 4:** Simulated changes in choice proportion of the NCD vaccine profiles.

Scenarios	Simulated changes in choice proportion		
	Vaccine profile 1 (Base = 59.68%)	Vaccine profile 2 (Base = 39.27%)	Opt-out (Base 1.04%)
Flock-level protection			
Profile1 = 70% efficacious	2.27	−2.18	−0.08
Profile2 = 70% efficacious	−1.78	1.88	−0.09
Delivery mechanism:			
Profile 1= by Veterinary technician	8.11%	−7.79	−0.32
Profile 2= by Veterinary technician	−6.82	7.02	-0.20

Route of administration			
Profile1=with water	3.95	3.73	−0.22
Profile2=with water	−5.23	5.45	−0.22
Profile1=with feed	−1.13	1.09	0.04
Profile2=with feed	1.85	−1.94	0.09

Bird-level protection			
Profile 1 = 60 percent of chicken would survive	17.27	−16.55	−0.71
Profile 2 = 60 percent of chicken would survive	−20.30	20.85	−0.55

## 4 Discussions

Newcastle disease (NCD) is considered the most important poultry disease worldwide and outbreaks of this disease can result in substantial mortalities among village poultry, affecting the livelihoods of poor farmers across developing countries. Innovations in the development of NCD vaccine technologies suitable for village poultry and implementation experiences in developing countries are encouraging. Designing a socially acceptable and economically sustainable NCD vaccine programme to control NCD in village poultry requires understanding farmers’ preference for attributes of possible vaccine programmes and the weight they would attach to attributes. The present research used a discrete choice experiment survey to investigate farmers’ preference for attributes of NCD vaccine programme in Ethiopia. The analysis of farmers’ preference for NCD vaccine attribute provides insights which may inform policy and future research on the design of NCD control efforts in village poultry and contribute towards efforts to improve poor farmers’ access to food.

The respective magnitudes of the parameter estimate of the RPL result convey important implication regarding the relative importance of attributes to respondents. The magnitude of the parameter estimates in our model showed that the most preferred attribute of a vaccine programme is the vaccine’s bird-level protection capacity to reduce mortality from NCD during an outbreak in terms of the proportion of chickens surviving the outbreak. This might be due to the fact that smallholder farmers in the study area occasionally lose a substantial proportion (at times 100%) of their poultry flock due to infectious poultry diseases in the event of NCD outbreak. Therefore, it is intuitive that farmers attach the highest weight for a NCD vaccine programme with higher bird-level protection capacity.

In the present production environment farmers are experiencing considerable chicken mortality and the weight attached to this vaccine attribute looks reasonable. However, reducing chicken mortality cannot be attributed only to NCD control, but chicken management, other poultry disease and nutrition also plays a role. Consequently, addressing farmers’ demand for a vaccine that has good capacity to reduce bird’s mortality through implementation of NCD vaccine may fail to achieve the intended results. This would negatively influence farmers’ perception about the vaccine and the likelihood for future vaccine technologies adoption and make extension systems more demanding. Therefore, it is important to carefully address all other chicken management issues together with vaccination to reduce chicken mortality which is primary concern for farmers.

The estimated parameters of the model result also indicate that the vaccine delivery mechanism, vaccine delivery by a veterinary technician, was the next most preferred attribute of an NCD vaccine. Although not assessing a specific animal health service (in contrast to the current research), Irungu et al. (2006) reported that community-based animal health workers were preferred to veterinarians and assistant animal health workers in Kenya due to their accessibility to farmers. In our study area, veterinary technicians (also called animal heath development agents) live in the village within the community and hence it was likely that accessibility was not a concern when farmers made their choices. It was also possible that farmers had limited confidence in trained farmers, as most farmers in the study area are illiterate. When providing animal health development agents’ services to every village is practically challenging, using community vaccinators may be a more realistic option. However, appropriate and adequate training is crucial to capacitate community vaccinators and build farmers’ confidence by giving equitable service. However, even with sufficient training, care may be needed to ensure farmers’ confidence in service providers, which is likely to greatly affect the uptake of the vaccine technology.

The third most preferred attribute was the route of administration. Farmers largely preferred and attached higher weight to NCD vaccine that could be given with water. This may suggest the need to consider acceptable routes of vaccine administration to ensure wider adoption of NCD vaccine technology by village poultry keepers. Village poultry NCD vaccine efficacy studies show that vaccines administered via water are efficacious compared with other roots (Thekisoe et al., 2004; Habibi et al., 2015). Therefore, future NCD vaccine programmes will have the potential to achieve a twin goal of achieving high efficacy and better acceptability as farmers highly prefer vaccines administered via water. The result also showed that farmers preferred and valued a vaccine service that could be given with water even more than the flock-level protection capacity of the vaccine. In the estimated model, respondents were found to display heterogeneous preferences for the attributes included in the study, particularly for the delivery mechanism and route of administration of the vaccine, suggesting the need to consider these features of the vaccine when extending poultry health interventions.

Flock-level protection from NCD was weighted less compared with other attributes; bird-level protection, vaccine delivery mechanism and route of administration. Farmers are usually experiencing sizeable losses of their chicken due to disease and, hence, are likely to be keen to ensure high bird-level protection. Hence, it is possible that they perceive reduction of mortality (bird-level protection) in the face of an NCD outbreak as a more realistic option than prevention of outbreaks themselves (flock-level protection).

Results from simulation of attribute levels to investigate how choices would change if the attribute levels of the NCD vaccine choices changed, supported the result from RPL estimation. Results from these simulations also revealed that bird-level protection capacity (70% in our case) and delivery of vaccine by animal heath extension enhances the acceptability of vaccine programme by farmers.

Generally, the important attributes of NCD vaccine programme in village poultry, ordered according to their weight to farmers, are: the vaccine’s bird-level protection capacity during an outbreak, the vaccine delivery mechanism, the route of vaccine administration and flock-level protection. It is, therefore, advisable to consider a range of important attributes of NCD vaccine programme in designing a policy to control NCD in village poultry so that any vaccine programme will be readily acceptable to farmers. This will help bring reasonable impact and help efforts to enhance livelihoods of farmers in developing countries that has similar production system.

This study has insightful findings, but with some limitations. Estimating monitory value of attributes was not possible as coefficient for price was insignificant. Simulation of choices by changing attribute levels was considered to address this limitation. Future researches may address this limitation by taking a wide range of price attribute levels in designing the choice experiment. Accounting for order of choice card presentations could also give an insight in future researches involving choice experiment in developing countries.

## 5 Conclusion

This study evaluates farmers’ preferences for features of possible NCD vaccine programmes. Results of the study revealed farmers prefer and prioritize NCD vaccine programme that has high capacity to protect birds from mortality. NCD vaccination programmes that would be administered via water and delivered by veterinary technician are preferred over those delivered by trained-peers. The results from estimates of RPL and the simulation results suggest the significance of understanding farmers’ preference for features of possible NCD vaccine programme to increase the acceptability of NCD control programme in village poultry. This will help in designing a NCD vaccine service that is more widely accepted and adopted across the country. This in turn is likely to enable farmers to fully realise the potential of village poultry sector that is currently limited by infectious poultry disease among other factors.

## Funding sources

This research work was supported by the UK Biotechnology and Biological Sciences Research Council (BBSRC), the UK Department for International Development (DFID) and the Scottish Government for Combating Infectious Diseases of Livestock for International Development (CIDLID) programme (BB/H009396/1, BB/H009159/1 and BB/H009051/1).

## Declarations of interest

None.

## Footnotes

1 Poultry production in Ethiopia almost solely involves chickens.
