# Supplementary material for: Eliciting preferences for attributes of Newcastle disease vaccination programmes for village poultry in Ethiopia
Source: Prev Vet Med. 2018 Oct 1;158:146–51. doi: 10.1016/j.prevetmed.2018.08.004 (PMC6152585; doi:10.1016/j.prevetmed.2018.08.004)
Supplement: Supplementary file 1 [file mmc1.pdf]

Sample choice cards used in DCE survey to elicit preference for attributes of Newcastle diseases vaccine

Block 2- Choice Set 5

Profile-1	Profile-2	Opt-out
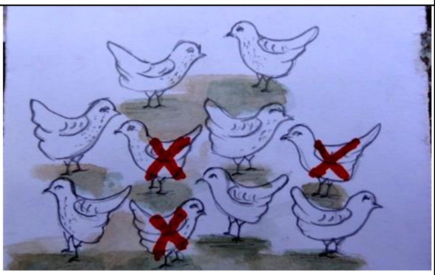<br>Flock-level protection - 70%	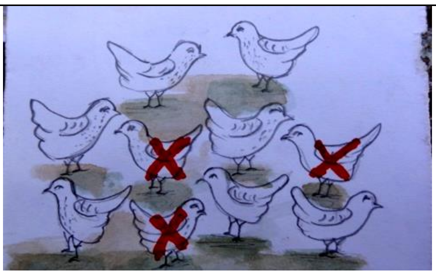<br>Flock-level protection - 70%	
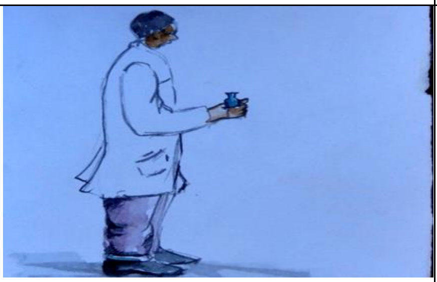<br>Delivery by: vet technician	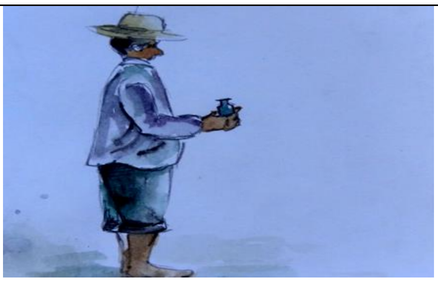<br>Delivery by: trained farmer	
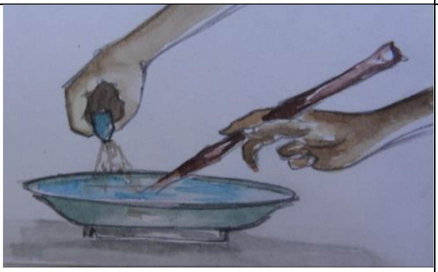<br>Route- with water	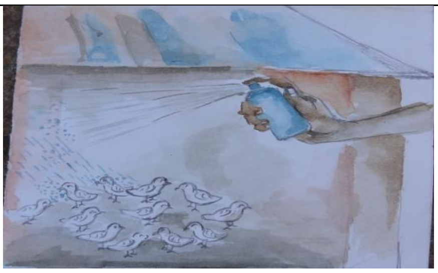<br>Route-Aerosol spray	
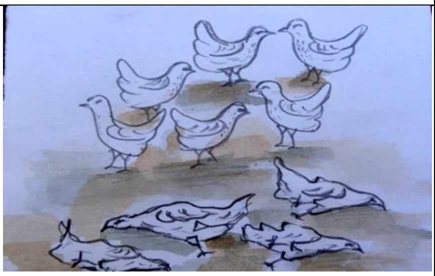<br>Bird-level protection - 60%	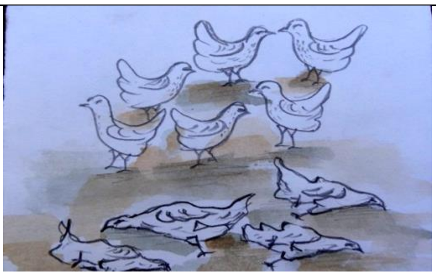<br>Bird-level protection - 60%	
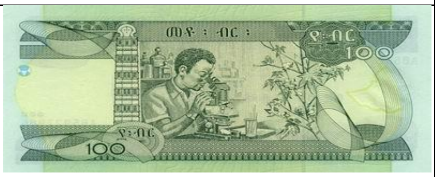<br>**Birr 100**	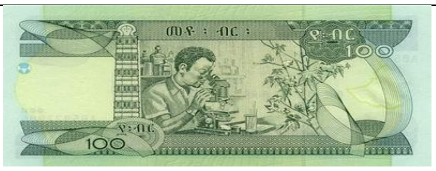<br>**Birr 100**	

Block 1- choice Set 4

Profile-1	Profile-2	Opt-out
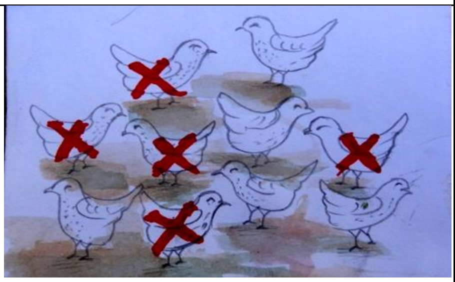 Flock-level protection - 50%	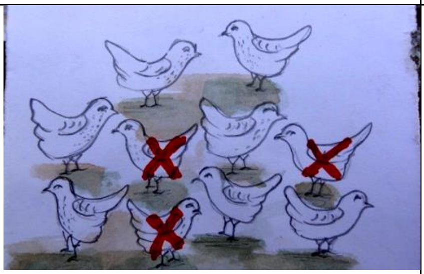 Flock-level protection - 70%	
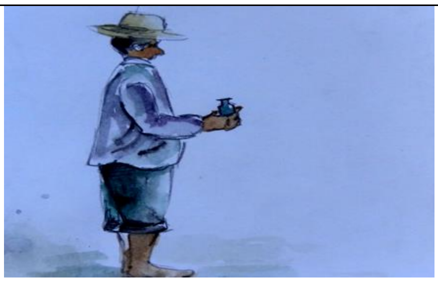 Delivery by: trained farmer	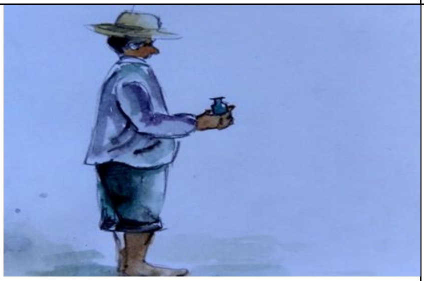 Delivery by: trained farmer	
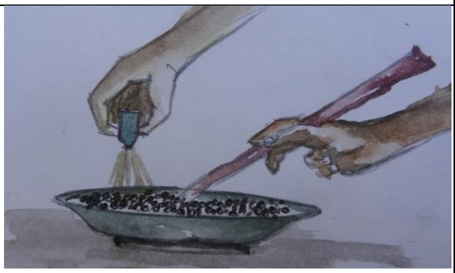 Route- with feed	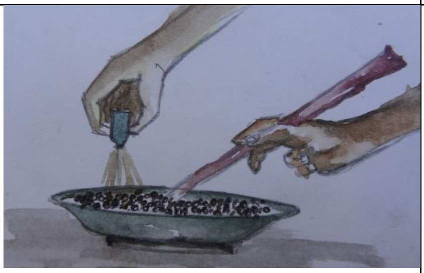 Route- with feed	
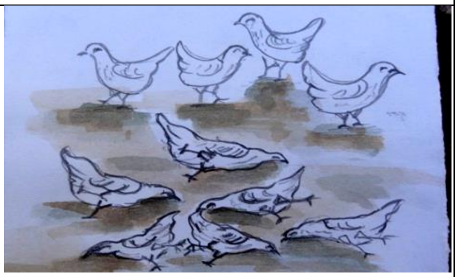 Bird-level protection - 40%	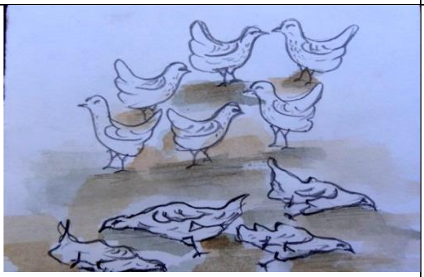 Bird-level protection - 60%	
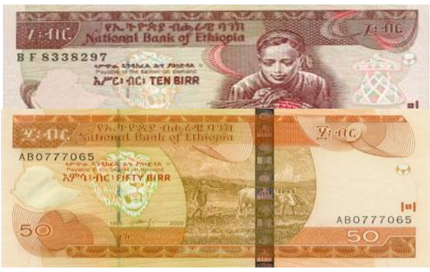 **Birr 60**	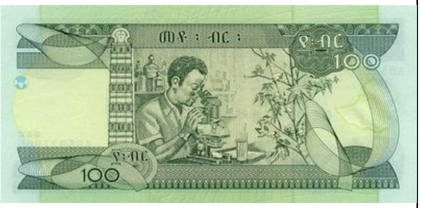 **Birr 100**	
